# Supplementary material for: Assessing transcriptomic reidentification risks using discriminative sequence models
Source: Genome Res. 2023 Jul;33(7):1101–12. doi: 10.1101/gr.277699.123 (PMC10538488; doi:10.1101/gr.277699.123)
Supplement: Supplement 2 [file Supplemental_Materials_Updated.pdf]

Supplemental Materials

Assessing transcriptomic re-identification risks using discriminative sequence models

Supplemental Figures

Supplemental Figure S1: Our hybrid EBL model outperforms the original EBL model

Supplemental Figure S2: DSM model-based p-values are lower than GNB and EBL on expanded GOT2D dataset

Supplemental Figure S3: DSM links a greater fraction of individuals on massive candidate genotype sets

Supplemental Figure S4: Memory requirement of DSM scales linearly in window and reference panel sizes

Supplemental Figure S5: Filtering to remove correlated eQTLs helps GNB and EBL models

Supplemental Figure S6: Graphical representation of DSM

Supplemental Tables

Supplemental Table S1: DSM's reverse linking is more accurate than linking based on predicted gene expression

Supplemental Notes

Supplemental Note S1: Model-based estimation of p-values

Supplemental Note S2: Evaluation metrics for linking with match score thresholds

Supplemental Note S3: Forward-backward algorithm for DSM

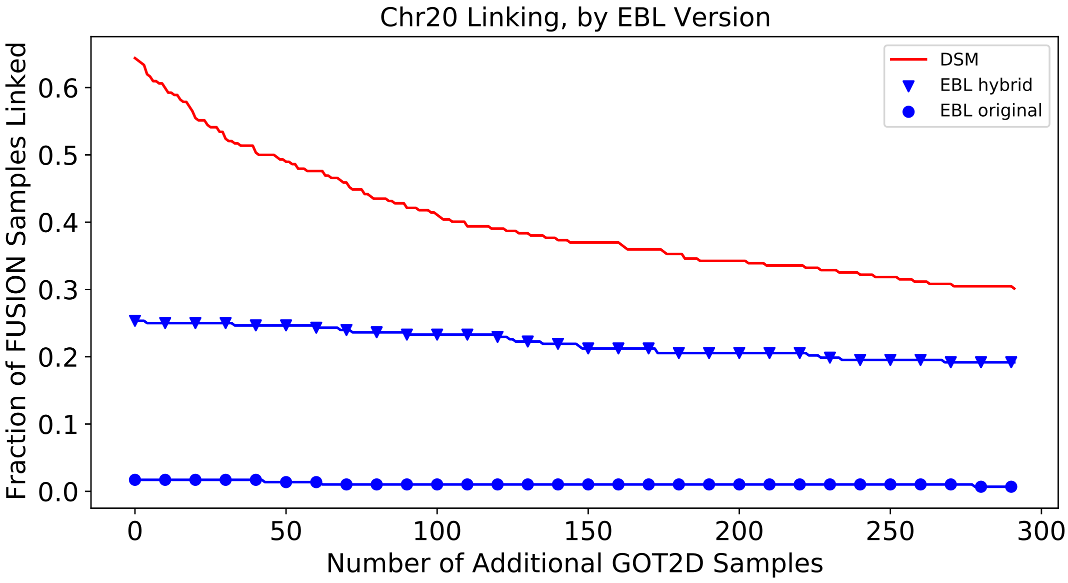

Supplemental Figure S1: **Our hybrid EBL model outperforms the original EBL model.** The original EBL models uses an uninformed prior $p(\mathbf{x})$ estimated from training data MAFs for those individuals and eQTLs where expression values are not extreme (circle). Using a hybrid approach enables a best-of-both-worlds scenario, in which eQTLs not associated to extreme expression values are still given an informative, non-uniform, posterior probability (triangle). The hybrid approach collapses to the GNB model for such eQTLs. Note that the original EBL model uses a sparse set of SNPs across the whole genome, which enables them to achieve high accuracy by using few highly informative SNPs. However, on a single chromosome there are unlikely to be a sufficient number of highly informative SNPs.

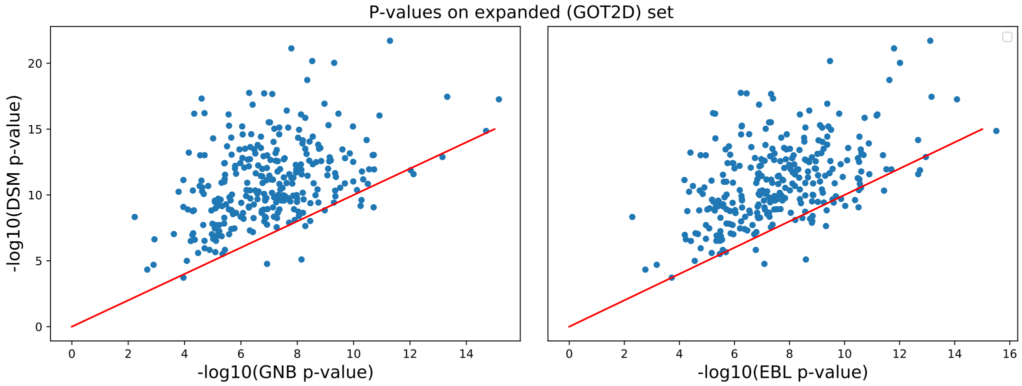

Supplemental Figure S2: **DSM model-based p-values are lower than GNB and EBL on expanded GOT2D dataset.** When including the expanded set of mismatching GOT2D genotypes for the matching score null distribution, we observe that DSM is able to consistently generate lower p-values than both previous approaches for all but a few individuals.

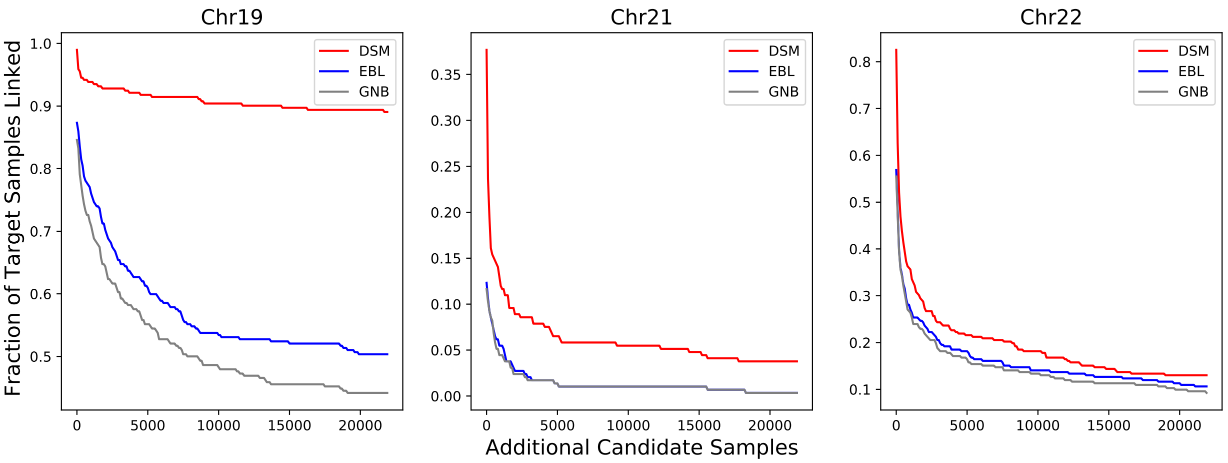

Supplemental Figure S3: **DSM links a greater fraction of individuals on massive candidate genotype sets.** On chromosomes 19, 21, and 22, we observed that DSM linked a substantially larger fraction of individuals when including ∼22k additional genotypes from HRC. This gap is largest in Chromosome 19, where DSM links >45% more individuals than EBL and GNB. The results for Chromosome 20 and for all four chomosomes combined are included in Figure 4.

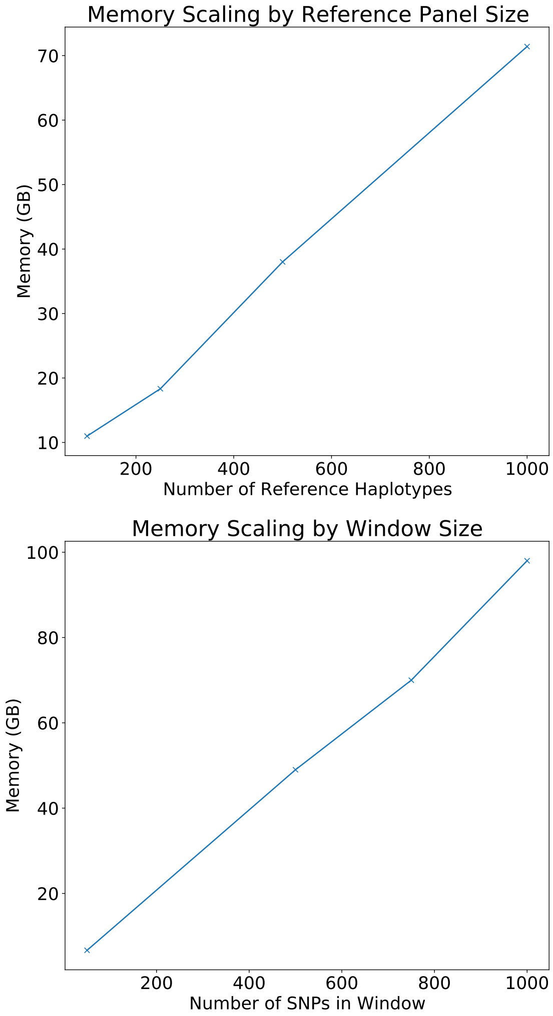

Supplemental Figure S4: **Memory requirement of DSM scales linearly in window and reference panel sizes.** Our haplotype-level factor approximation enables us to use a far larger reference panel without quadratic memory requirements (**top**). We also enable linear scaling in memory in the size of windows considered (**bottom**). In practice, we used a reference panel of 1000 haplotypes and window size of 750 eQTLs, which allowed us to train the DSM with a practical memory requirement of ∼75GB. These are notably larger numbers than those required for GNB and EBL, which can each be done on the order of minutes for a full chromosome with less than 5GB memory. However, note that it is reasonable to consider an adversary with sufficient computational resources for the purpose of assessing the risk of linking attacks.

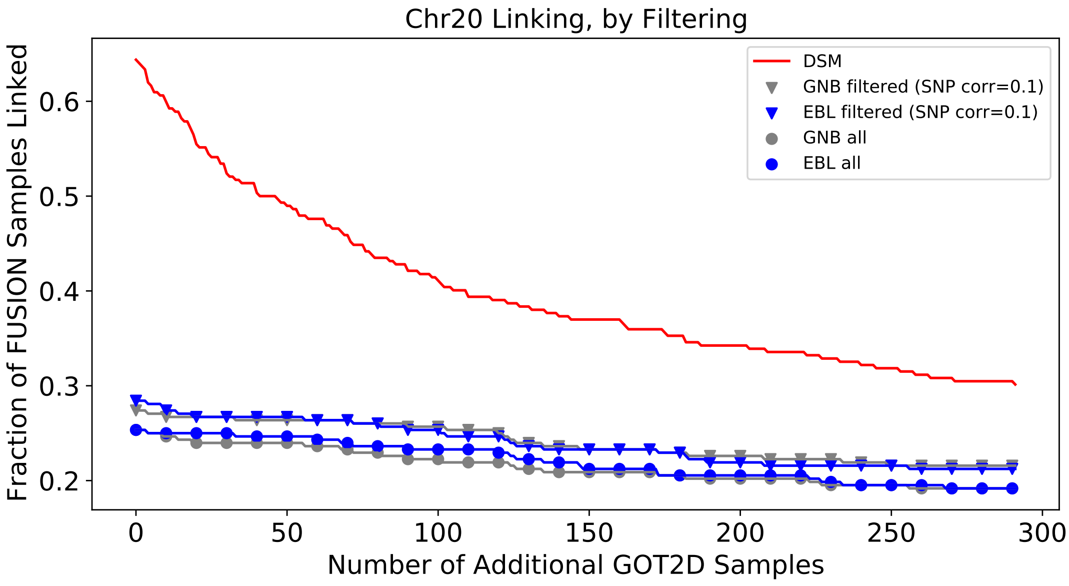

Supplemental Figure S5: **Filtering to remove correlated eQTLs helps GNB and EBL models.** EBL and GNB both assume some level of pairwise independence between eQTLs, as including all eQTLs can lead to miscalibrated matching score probabilities. For both these models, the linking accuracy pre-filtering (circle) is worse than the linking accuracy post-filtering (triangle). The SNP set is greedily pruned until no two SNPs have pairwise correlation greater than 0.1.

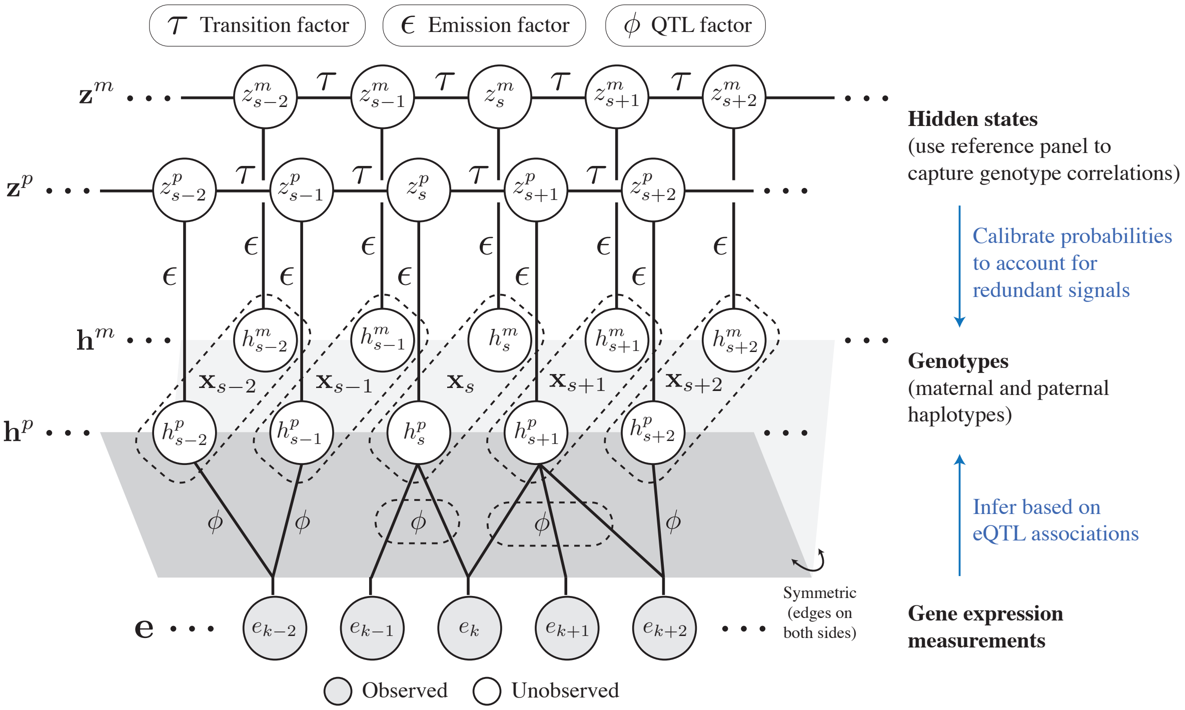

Supplemental Figure S6: **Graphical representation of DSM.** Solid edges indicate factors in the conditional distribution $p(\mathbf{x}|\mathbf{e})$, including transition, emission, and QTL factors. $\mathbf{h}$ corresponds to the haplotype, of which there are two (together denoted $\mathbf{x}$, indicated by dotted lines), while $\mathbf{e}$ corresponds to the expression levels of individual genes. The ϕ factors, some of which involve multiple genes (dotted lines), relate predictive signals in the gene expression data to the genotypes (pairs of alleles). To enable the use of large reference panels, we predict the haplotype allele and copy this prediction twice (gray foldout). The τ and ϵ factors respectively correspond to the recombination and emission probabilities in the standard Li-Stephens model. $\mathbf{z}^m$ and $\mathbf{z}^p$ index into the reference panel of haplotypes, whose posteriors, conditioned on observed gene expression values, are updated via the forward-backward algorithm.

Chromosome	MetaXcan (Pearson)	MetaXcan (Spearman's)	DSM
19	114 (39.0%)	51 (17.5%)	**291** (99.7%)
20	15 (5.1%)	6 (2.0%)	**240** (82.2%)
21	3 (1.0%)	4 (1.4%)	**157** (53.8%)
22	24 (8.2%)	13 (4.5%)	**263** (90.0%)
All 4 combined	171 (58.6%)	97 (33.2%)	**291/292** (99.7%)

Supplemental Table 1: **DSM's reverse linking is more accurate than linking based on predicted gene expression.** We report DSM's accuracy in linking genotype profiles to matching expression profiles (i.e., in the reverse direction from our primary evaluation setting), compared to an alternative approach based on a state-of-the-art gene expression prediction method, MetaXcan [Barbeira et al., *Nature Communications*, 2018]. The evaluations are performed on the FUSION dataset. We used the pretrained MetaXcan model on GTEx v8 muscle-skeletal tissue dataset, the same dataset used to train DSM. Both Pearson and Spearman's correlation coefficients between the predicted and observed expression profiles are considered as the match score for MetaXcan. DSM leads to more accurate linking than matching based on the predicted gene expression levels from MetaXcan.

Supplemental Note 1: Model-based estimation of p-values

Let $\mathbf{M} = [M_1, \cdots, M_N]$ be a sorted vector of match scores calculated for a particular individual, and let M^* be the score for the true match. When the true match score is the largest match $M^* = M_N$, then the individual is correctly linked. In this case, we wish to calculate a measure of how strong this identifying signal is based on the relative magnitude of M^* compared to the rest of the match scores in $\mathbf{M}$. We achieve this by estimating a p-value based on a parameterized null distribution that is fit to the non-matching samples in $\mathbf{M}$. We use a Gaussian distribution to model the null distribution, after empirically observing the approximate normality of the null distributions.

For robust estimation of the model parameters, we trim both tails of the empirical distribution (also excluding the true match) before estimating the mean and the variance of the distribution. For instance, the mean is estimated as

$$\mu^{\mathsf{trimmed}} = \frac{1}{N - 2k - 1} \sum_{i=k+1}^{N-k} M_i \cdot \mathbf{1}\{M_i \neq M^*\},$$

where $\mathbf{1}\{\cdot\}$ is an indicator function, and k determines the amount of trimming ($k/N = 0.2$ in our experiments). The standard deviation $\sigma^{\mathsf{trimmed}}$ is similarly estimated using this trimmed distribution, then scaled to obtain an unbiased estimator using the theoretical quantity from the corresponding truncated Gaussian distribution.

The z-score of our true match M^* is thus

$$z^* = \frac{M^* - \mu^{\mathsf{trimmed}}}{\sigma^{\mathsf{trimmed}}}.$$

The p-value is calculated by first taking the tail probability of z^* in the standard normal distribution. We then re-weight the p-value obtained from the z-score by multiplying by a weight w, defined as

$$w = \frac{2}{N - 1} \sum_{i=1}^{N} \mathbf{1}\{M_i > \mu^{\mathsf{trimmed}}\} \cdot \mathbf{1}\{M_i \neq M^*\}.$$

This represents two times the proportion of match scores that lie above the trimmed mean, which corrects for potential asymmetry in the null distribution below and above the mean. Empirically, we observe that our above approach leads to accurate model-based p-values that are consistent with permutation-based, empirical p-values (see Fig. 4C).

Supplemental Note 2: Evaluation metrics for linking with match score thresholds

To investigate an attack scenario in which the attacker must make a decision about whether the proposed match is a true match, we evaluated the full range of match score thresholds for each of the three methods with respect to a number of standard metrics for binary classification. We extended these metrics to the setting of linking attacks. The key difference in our setting is that some data instances may never be linked correctly regardless of the threshold, if an incorrect match with a higher match score exists.

For each trial of our holdout experiment, we randomly split the individuals in our test dataset (FUSION) into two halves, which we term Set 1 and Set 2. We keep the genotype profiles of Set 1 individuals in the target genotype set, while excluding them for Set 2 individuals in order to assess the rate of false matches for individuals who are not present in the target set. For each choice of the match score threshold, we compute precision, recall (true positive rate), and false positive rate as follows.

Let n_1 and n_2 be the number of individuals in Set 1 and Set 2, respectively. For Set 1, let TP_1 ("true positives") or FP_1 ("false positives") be the number of individuals who are correctly or incorrectly linked, respectively, with a match score above the threshold. For Set 2, let FP_2 ("false positives") be the number of individuals who were incorrectly linked with a match score above the threshold. Note that all individuals in Set 1 are technically considered "positives" since there exists a true match in the target set. Individuals represented by FP_1 are an exception, who are positives that are converted to negatives due to problematic match scores. All individuals in Set 2 are considered "negatives" since a true match does not exist. Given these terms, we calculate

$$\begin{aligned}
\text{Precision} &= \frac{\mathrm{TP}_1}{\mathrm{TP}_1 + \mathrm{FP}_1 + \mathrm{FP}_2}, \\
\text{Recall or True Positive Rate (TPR)} &= \frac{\mathrm{TP}_1}{n_1}, \\
\text{False Positive Rate (FPR)} &= \frac{\mathrm{FP}_2}{n_2}.
\end{aligned}$$

For FPR, we do not consider FP_2 to keep the denominator of n_2 fixed across different methods.

The receiver-operating-characteristic (ROC) and precision-recall (PR) curves based on the metrics above are reported in Fig. 5. For each split and method, we interpolate PR curves from 0 to the maximum recall on the x-axis for that particular split. The ROC curve is similarly interpolated by considering all FPRs from 0 to 0.99.

We note that our modified metrics for linking performance result in ROC and PR curves that do not end at the typical end points, i.e., (1,1) and (1,0), respectively. This is because there are certain individuals (FP_1) who are incorrectly classified regardless of the decision threshold since the score for the true match is less than the score for the top match for these individuals.

The AUPRC and AUROC metrics reported for each curve considers an x-axis threshold α up to which the area under the curve (AUC) is computed and then rescaled by $\frac{1}{\alpha}$. We adopt this truncation approach to equalize the comparisons across methods, given that the location of the end point of each curve (corresponding to a decision threshold that obtains the maximum recall) is different across methods due to the FP_1 issue describe above. For AUROC, we chose $\alpha = 0.97$ as the FPR threshold and for AUPRC, we chose $\alpha = 0.85$ as the recall threshold, based on the lowest end point of the curves.

Supplemental Note 3: Forward-backward algorithm for DSM

Recall that during inference, we wish to calculate

$$M(\mathbf{x}, \mathbf{e}) := p_{\mathsf{DSM}}(\mathbf{x}|\mathbf{e}).$$

Our haplotype-level approximation factorizes this term as:

$$p_{\mathsf{DSM}}(\mathbf{x} = (\mathbf{h}^m, \mathbf{h}^p) \mid \mathbf{e}) \approx p_{\mathsf{DSM}}(\mathbf{h}^m|\mathbf{e}) p_{\mathsf{DSM}}(\mathbf{h}^p|\mathbf{e}),$$

where

$$p_{\mathsf{DSM}}(\mathbf{h}^m|\mathbf{e}) \propto \sum_{\mathbf{z}^m} \tilde{p}_{\mathsf{QTL}}(\mathbf{h}^m, \mathbf{e}) \tilde{p}_{\mathsf{HMM}}(\mathbf{h}^m, \mathbf{z}^m),$$

and analogously for $\tilde{p}_{\mathsf{DSM}}(\mathbf{h}^p|\mathbf{e})$. Note that $\tilde{p}_{\mathsf{QTL}}$ represents (unnormalized) probabilistic factors capturing eQTL associations, and $\tilde{p}_{\mathsf{HMM}}$ represents HMM factors—i.e., transition and emission probabilities over the genotypes and hidden states only.

Here we describe a forward-backward algorithm for computing $\tilde{p}_{\mathsf{DSM}}(\mathbf{h}^m|\mathbf{e})$, which is the same as that for $\mathbf{h}^p$. First, note that

$$p_{\mathsf{DSM}}(\mathbf{h}^m|\mathbf{e}) = \prod_{j=1}^{V} p_{\mathsf{DSM}}(h_j^m|\mathbf{h}_{<j}^m, \mathbf{e})$$

by chain rule, where $\mathbf{h}_{<j}^m$ denotes all genotypes that precede the j-th position. Each term can be computed using the following relation based on the independence structure of DSM:

$$p_{\mathsf{DSM}}(h_j^m|\mathbf{h}_{<j}^m, \mathbf{e}) = \sum_{z_j^m} p_{\mathsf{DSM}}(h_j^m, z_j^m|\mathbf{h}_{<j}^m, \mathbf{e}) \propto \sum_{z_j^m} \tilde{p}_{\mathsf{HMM}}(z_j^m|\mathbf{h}_{<j}^m) \tilde{p}_{\mathsf{HMM}}(h_j^m|z_j^m) \tilde{p}_{\mathsf{QTL}}(h_j^m, \mathbf{e}_{Q(j)}) \tilde{p}_{\mathsf{DSM}}(\mathbf{e}_{Q(>j)}|z_j^m).$$

We denote the set of eGenes associated the j-th eQTL as $\mathbf{e}_{Q(j)}$ and those associated with any of the subsequent eQTLs as $\mathbf{e}_{Q(>i)}$. Our modification of the standard forward-backward algorithm therefore calculates the "forward" probability, i.e., $\tilde{p}_{\mathsf{DSM}}(z_j^m|\mathbf{h}_{<j}^m)$, and the "backward" probability, i.e., $\tilde{p}_{\mathsf{DSM}}(\mathbf{e}_{>j}|z_j^m)$, for all j via dynamic programming. The forward term is analogous to the Li-Stephens model, whereas the backward term newly incorporates information from the observed gene expression. Once these terms are computed, the above equation can be used to compute the full conditional likelihood $p_{\mathsf{DSM}}(\mathbf{h}^m|\mathbf{e})$ as desired.

More detailed computational steps of the forward-backward algorithm are as follows. We begin by defining a dynamic programming matrix $\alpha \in [0,1]^{V \times R}$, where V is the number of eQTLs and R is the number of reference haplotypes, that stores the (unnormalized) forward probabilities of z_j^m for each eQTL j. Our initial belief over z_1^m,

the hidden state for the first eQTL, is uniformly initialized:

$$\alpha(1, z) = 1$$

for all $z \in \{1, \ldots, R\}$. Then, sequentially for each $j \in \{2, \ldots, V\}$, we compute

$$\alpha(j, z) = \sum_{z' \in \{1,\ldots,R\}} \alpha(j-1, z') \cdot \tau(z|z') \cdot \epsilon(h_{j-1}^m|z).$$

Here, τ and ϵ indicates the transition and emission probabilities of the HMM component of DSM, respectively. After each step, we rescale $\alpha(j, z)$ to prevent an underflow.

For backward probabilities, we define a dynamic programming matrix $\beta \in [0, 1]^{V \times R}$ that stores probabilities over z_j^m for each eQTL j. Specifically, we begin by setting $\beta(V, z) = 1$ for all $z \in \{1, \ldots, R\}$. Then, for all $j \in \{V-1, \ldots, 1\}$, we set:

$$\beta(j, z) = \sum_{z' \in \{1,\ldots,R\}} \beta(j+1, z') \cdot \tau(z'|z) \cdot \left[\phi_j(h_j^m, \mathbf{e}_{Q(j)}) \cdot \epsilon(h_j^m|z) + \phi_j(1 - h_j^m, \mathbf{e}_{Q(j)}) \cdot \epsilon(1 - h_j^m|z)\right],$$

where ϕ_j indicates the eQTL factor at position j, which we introduced to model the association between each eQTL and a corresponding set of eGenes.

We also note that while the transition factor $\tau(z|z')$ is naïvely quadratic in the number of reference haplotypes, $\tau(z|z')$ has a structured matrix form:

$$\tau(z|z') = \begin{cases} 1 - \rho + \frac{\rho}{R}, & \text{if } z = z', \\ \frac{\rho}{R}, & \text{if } z \neq z', \end{cases}$$

where ρ is the recombination rate between the two eQTLs. Thus, the matrix is symmetric and constant everywhere but the diagonal, which enables an efficient multiplication with the transition matrix, linear in the number of reference haplotypes.

The computational cost of the above computation is $O(R)$ for each of V eQTLs, resulting in the overall complexity of $O(VR)$. Our memory requirement also has the same complexity, since the sizes of α and β are both $O(VR)$. Thus, our runtime and memory grow linearly in both the number of eQTLs (window size) and the number of reference haplotypes. We provide empirical results illustrating the linear memory scaling of our model in Supplemental Fig. S4.
